# Suicide amongst the Inuit of Nunavut: An Exploration of Life Trajectories

**DOI:** 10.3390/ijerph17061812

**Published:** 2020-03-11

**Authors:** William Affleck, Eduardo Chachamovich, Nadia Chawky, Guy Beauchamp, Gustavo Turecki, Monique Séguin

**Affiliations:** 1 Department of Psychiatry, McGill University, Montréal, QC H3A 0G4, Canada; eduardo.chachamovich@douglas.mcgill.ca (E.C.); gustavo.turecki@mcgill.ca (G.T.); 2 Département de psychoéducation et psychologie, Université du Québec en Outaouais, Gatineau, QC J9A 1L8, Canada; 3 McGill Group for Suicide Studies, Montréal, QC H3A 0G4, Canada; Nadia.chawky@gmail.com (N.C.); yowawa95@gmail.com (G.B.); Monique.Seguin@uqo.ca (M.S.)

**Keywords:** suicide, Inuit, life trajectory

## Abstract

This article reports results of the life trajectories from 92 Inuit who died by suicide, matched for age and gender with 92 living-controls. A proxy-based procedure and semi-structured interviews with informants were conducted to obtain trajectories of developmental events occurring over the life course for suicide and community-matched controls. Results from this research indicate two different trajectories that differentiate the control-group from the suicide-group throughout the life course. Even though the number of suicide attempts are similar between both groups, the suicide-group had a more important burden of adversity, which seemed to create a cascading effect, leading to suicide.

## 1. Introduction

Inuit are a group of culturally similar Indigenous peoples inhabiting the Arctic regions of Greenland, Canada, and the United States. Approximately 65,000 Inuit live in Canada. Of these, the majority live in the Inuit homeland of Inuit Nunangat, which is comprised of four Inuit regions: Nunavut, Nunavik, Nunatsiavut, and the Inuvialuit Settlement Region [1].

Inuit in Canada currently suffer from one of the highest rates of suicide in the world [2,3,4]. Rates range from five to 25 times the Canadian average. In the five-year period, from 2009–2013, Canada’s suicide rate was 11 per 100,000. In *Inuit Nunangat*, rates ranged from 60 per 100,000 in the *Inuvialuit Settlement Region* to 275 per 100,000 in *Nunatsiavut* [5]. Rates are highest for young Inuit men, who are 40 times more likely to die by suicide than their peers in Southern Canada [6].

Theories of suicide have converged to identify major risk factors implicated in the complex etiology of suicide [7]. Models suggest that distal predisposing variables (i.e., temperament and genetics) and developmental variables (i.e., early adversity and cognitive style) impact upon proximal or precipitating factors, such as mental health problems and substance abuse, which when coupled with recent life events, can increase vulnerability to suicide [8,9,10,11,12,13,14,15].

In Western populations, theories of suicide are most often based on individual suffering, as opposed to Indigenous populations, where suicide is largely considered as social suffering [16]. Research on Indigenous populations, including the Inuit, has tended to focus on larger systemic factors that resulted from colonization to account for the high rates of suicide [17]. From this perspective, suicide is viewed not only as an individual problem but also as an outcome of socioeconomic marginalization; loss of culture and language; and government policies and practices that separated families and forced assimilation through residential schools, where widespread abuse and violence had profound effects on individuals and communities [18,19,20,21,22,23,24,25]. Identified risk factors for suicide amongst the Inuit reflect this understanding, and they include historical trauma, social iniquities, intergenerational trauma, the loss of culture and language, and mental distress and illness [4].

Developmental research has introduced the concept of cumulative advantage/disadvantage, suggesting that early life events pave the road to an individual’s future health and well-being [26,27]. The presence of early adversities related to family structure, abuse and trauma, and relational and social difficulties may have distinct long-term effects because they impede on an individual’s development in different domains, such as interpersonal, social, professional, and health issues [28,29].

Our research group has been using a life course approach to identify different pathways leading toward and away from suicide [30,31]. In an earlier study, we outlined the role that protective factors play in the prevention of suicide for Inuit across the life course [32]. To our knowledge, no other study has investigated the sequence of life events and the different developmental experiences of Inuit peoples. The current study investigated individual trajectories in order to identify the presence and chronology of major adversity in the lives of Inuit in Nunavut (suicide and living control). A better understanding of the interaction between individual vulnerabilities and contextual or environmental risk factors can aid policy makers in developing targeted suicide-prevention interventions. 

## 2. Materials and Method

### 2.1. Qaujivallianiq Inuusirijauvalauqtunik (“Learning from Lives that have been Lived”)

From 2005 to 2010, a large follow-back study on suicide, an age- and gender-matched case-control study, was conducted among the Inuit of Nunavut by investigating all suicide cases that occurred in the territory between 2003 and 2006 [33,34]. Living-controls were selected from the Nunavut Health Care Registration File. For each suicide, the researchers randomly selected a community control that matched on gender and age. The study used two different types of interviews, both using a proxy-based interview procedure to gather information on mental health problems, life events, protective factors, and adversities: First, we used the psychological autopsy method, which consists of interviewing close relatives and/or friends of the deceased, using a combination of open-ended questions and standardized instruments to gather information on the deceased [35]. Second, we used the life calendar trajectory method, an interview technique in which life events are mapped onto different developmental spheres of an individual’s life trajectory [31].

From the initial 240 clinical vignettes completed by the research team for data on mental health problems, 92 suicide cases had sufficient information to analyze the life trajectory. This study reports on the life trajectory of these 92 suicide cases and 92 matched living-controls. The number of informants necessary to complete the interview was determined by the interviewer, based on the quality of the interviews and the amount of information gathered. For this study, 186 interviews were conducted for suicide cases (mean of 2 interviews per case), and 148 interviews for controls (mean of 1.6 interview per case) were carried out. Respondents for the suicide group and controls respectively were parents (54 vs. 42), partners (19 vs. 28), siblings (29 vs. 40), extended family members (43 vs. 15), friends (35 vs. 23), and professionals (6 vs. 0) for the suicide group and controls, respectively. All interviews were conducted in English or Inuktitut, according to the respondent’s preference. Medical charts, coroner’s notes, and criminal records were also systematically reviewed.

The Institutional Research Ethics Board (REB) of the Douglas Mental Health University Institute approved the study, and the Nunavut Research Institute issued a research license. The project was developed in partnership with community organizations in Nunavut, including the regional suicide prevention organization, the Embrace Life Council and Nunavut Tunngavik Incorporated (NTI) [34]. Results from this study were presented to stakeholders and community members before publication, as required by chapter 9 of the second edition of the Tri-Council Policy Statement: Ethical conduct for Research Involving Humans [36]. The first author met on numerous occasions with representatives of NTI in Iqaluit, Nunavut, who approved both the results and the language used in the article.

### 2.2. Life-Trajectory Methodology

The life calendar method was embedded in the larger study [33,34]. The current paper focuses on results from the life-trajectory interviews, which investigated the occurrence of life events and the cumulative burden of adversity over the life course. This interview method, which has been described elsewhere [30,31], uses a life trajectory calendar to reconstruct the major events of an individual’s life.

The interview uses a narrative approach in order to elicit information relevant to understand the context in which the significant life events occurred. Each life event may be perceived differently and may have different consequences depending on the circumstances in which it takes place. For example, birth, adoption, and marriage may have distinct consequences depending on the culture and the individual contexts in which they occur [37]. Some events, like custom adoption, which is common in Inuit culture, may have specific consequences for the individual and others in their social environment [38]. Using a context-sensitive method of inquiry helps establish a distinction between events, especially in light of different cultural norms. The different life spheres discussed during the life-trajectory interview were as follows: circumstances of death (method, motivation, and intoxication by alcohol/drugs); place of residence and housing; experiences during childhood and adolescence; interpersonal and romantic relationships; relations with friends; school and academic experiences; professional experiences; legal problems; family history of suicide and other family adversities; personal difficulties and mental health problems; and mental health services received.

The interviews started with general questions, such as the following: “Could you tell me how (name of case or control) was as a child?” and “Could you tell me about (name’s) school attendance?” Interviewers would then probe further and ask detailed questions to verify if particular events happened or not in specific spheres of the individual’s life course. Each sphere included a number of variables that were important to investigate. Since more than one informant was interviewed, the multiple narrative accounts from different perspectives enriched the information pertaining to the occurrence and the context of each life event. Using this narrative methodology [38], interviewers sought to accumulate sufficient details about the individual’s life events and its trajectory to allow trained evaluators to assess the key characteristics of each event, for example, duration, severity, and frequency.

### 2.3. Psychological Autopsy Method

Mental health and personality disorders were assessed by administering the Structured Clinical Interview for DSM-IV Axis I and Axis II [39,40]. These results have already been reported [33]. In this paper, we report the age in which mental health disorders occurred and their durations.

### 2.4. Sociodemographic Variables

Finally, sociodemographic variables were assessed by using standardized instruments culturally adapted to Nunavut [33].

### 2.5. Procedure and Analysis

The first step was to identify and code each event, the age period, and its duration in a graphic calendar. When the interviews with participants were completed, clinical case histories (case vignettes) were drafted with the information obtained from all the measurements and submitted to the panel of evaluators who were independent from the interviewers. The panel was composed of researchers from our team, clinical practitioners, and psychologists who analyzed the life trajectories and established a summary variable identified as the “overall contextual burden of adversity rating” for every 5-year interval of the life trajectory. The evaluators assessed the “contextual burden” of events by assessing their relative weight within the respondent’s developmental circumstances. This conceptualization of contextual burden was borrowed from the morbidity burden or low disease burden approach used to identify the overall morbidity that affects health. It is associated with allostatic load, a concept that links psychosocial stress with the neurobiological and genetic dimensions of mental disorders and suicide. The overall burden assessments ranged from severe (rating of 5 or 6) to moderate (3 or 4) to low (1 or 2). Case reference logs were written and used to maintain the same evaluation across all cases. For all cases, the evaluators coded the burden of adversity for each 5-year period independently before reaching a consensus through discussion. In previous studies from our research group, the intra-pair agreement for each 5-year segment ranged from 76% to 97%; the lowest agreement was found in the 0-to-4-year’s age segment.

**Figure 1:**
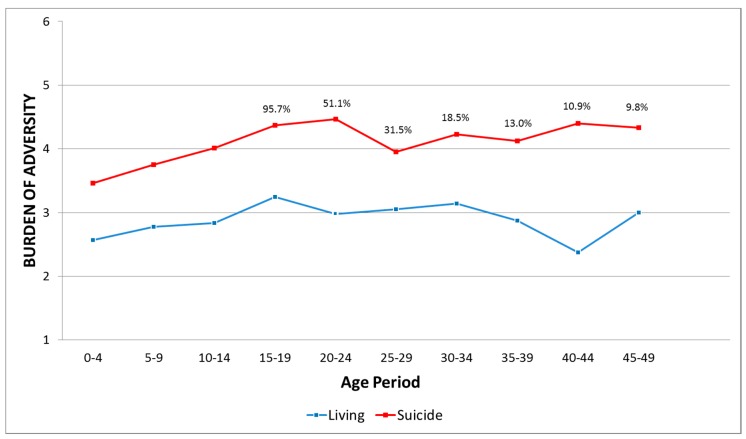
Trajectories of control-group and suicide cases. The % corresponds to the proportion of serving individuals on entry of the age period for the suicide group.

The second step was to study the burden of adversity over time. Combined Discrete Time Survival (DTS) and Growth Modeling (GM), using the software Mplus Version 7.4 for Mac [41], were used to examine the individual variation in burden of adversity within age periods (0–4 years through to 45–49 years) for control and suicide cases in a multigroup analysis. GM can identify distinct sets of continuous growth factors, which are indicated by the intercept, slope, and quadratic term. Suicide is considered as a unique event in time, and in that respect, DTS analysis (generating proportional odds continuous latent variable) was added to the GM. The complete statistical model is illustrated in Figure 1. The objective of this method of analysis was to examine the pattern of variation and stability over time for each group.

The third step was identifying the presence of cumulative adversity and pathways to suicide. Each life calendar was analyzed individually, and the number of events in each 5-year age period was recorded. Based on clinical vignettes, we organized the data into four different life spheres: (1) early adversity of maltreatment; (2) household and family dysfunction; (3) personal/mental health difficulties; and (4) social difficulties. In each sphere, we grouped a number of variables that were identified by respondents during the interview process. Early adversity of maltreatment included four variables: familial sexual abuse, sexual violence, physical violence, and psychological violence. The sphere of *household and family dysfunction* included the possible presence of 16 variables: incoherent rules, lack of discipline, lack of supervision, tension with parents, negligence, role reversal, affective distance, family secrets, parental separation, parental divorce, parental mental health difficulties, witnessing parental violence, living with someone who misused alcohol, tension with sibling, food insecurity, and adverse housing conditions. *Personal/mental health difficulties* included the possible presence of nine variables: Axis I diagnosis (depression, anxiety, gambling, and schizophrenia), alcohol and drug abuse, personality disorder, number of suicide attempts, self-harm, and psychiatric hospitalization. *Social difficulties* included the possible presence of five variables: conflict with peers, social isolation, bad influence from peers resulting in legal problems, separation or loss of a friend, and difficulty in romantic relationship or commitment.

## 3. Results

### 3.1. Sociodemographic

A total of 184 life calendars were analyzed: 92 pairs (suicide and living controls) matched for date of birth and gender (14 women in each group). Analysis of the sociodemographic data identified differences between the two groups. Individuals in the living control group were on average older (Mean Age = 27.8, *SD* = 9.0) than the suicide group (Mean Age = 23.2, *SD* = 9.0) (*t* (182) = 3.48). Results indicate that 49% of individuals in the suicide group died before 20 years of age; by 25 years of age, 68.5% had died, and by 30 years of age, 81.5% had died. Reflecting the group difference, there was a greater proportion of living controls with a married status (46.7% vs. 25.0%, chi-square = 8.5, *p* < 0.005), and a job (66.3% vs. 35.9%, chi-square = 15.9, *p* < 0.001). There was a significantly greater proportion of individuals with a high school diploma in the control group compared to the suicide group (23 (25.0%) vs. 6 (6.5%), chi-square =10.5, *p* < 0.005).

### 3.2. Comparison between Group Trajectories

The longitudinal burden of adversity (summary variable) data were analyzed in order to compare the global burden of adversity of each group, for every five-year period. The shape of the two trajectories and their continuous growth factors (Figure 1) exhibited two different trajectories pertaining to suicide or living controls. The x-axis corresponds to the age period and the y-axis to the burden of adversity (low burden 1 or 2; moderate burden 3 or 4; and high burden 5 or 6). Based on this figure, both groups have a somewhat parallel but distinct burden of adversity throughout their lives. Individuals in the living control group were essentially exposed to overall moderate adversity, which slightly increased over time (Table 1, significant linear term). The significance of the quadratic terms is due to the increase in the burden of adversity taking place in the 40 to 44 age period for this group. Individuals in the suicide group had a larger overall burden of adversity score during their lifetime, varying from moderate to severe, whereas the most severe period was between 14 and 25 years, with a slightly descending overall burden after 25 years (Table 1).

These two different trajectories in regard to the burden of adversity are carried out throughout the life course. Results of the presence of early maltreatment during the five-to-nine age period indicated that 33.7% of children in the suicide group were subjected to one, two, or three types of violence during the five-to-nine age period, compared to 17.4% in the control group (Table 2). However, psychological violence was the only significant variable between the suicide group and the control group (20.7% vs. 7.6%).

As for the number of events present for each sphere, all demonstrate significant results (Table 3). The data indicate how many events were present in the lives of individuals, in both groups, and the *zero %* gives the proportion of individuals who were not exposed to any events registered to a specific sphere. For example, results for the first sphere, *early adversity of maltreatment*, indicate that 66% of individuals who died by suicide were not subjected to early maltreatment and violence, compared to 78% in the living-control group. In the second sphere, *household and family dysfunction*, 14% of individuals from the suicide group were not exposed to any adversities, compared to 33% for living-controls. As for the third sphere, *personal and mental health difficulties*, 53% of those in the suicide group were not exposed to personal difficulties (mental health and substance misuse) during the 10–14 age-year period, compared to 83% of the control group. Finally, in the sphere of *social difficulties*, 75% of individuals in the suicide group were not exposed to social difficulties during the 15–19 age period, compared to 89% of the control-group. Thus, there were significant differences between groups in all four spheres. 

The data of all Axis I diagnosis were merged together at age 5–9, 10–14, and 15–19, and Axis II diagnosis was merged after 19, in order to determine the importance of this variable on suicide (see Table 4). Alcohol and drug abuse were included as a specific disorder—and not added in Axis I— because it was significant by itself, especially after 10 years of age. Results show that the prevalence of mental health problems was greater for those in the suicide group compared to those in the control group during the 10-to-14 age period with regard to Axis I disorders and alcohol and drug abuse. For the 15-to-19 age period, the suicide group had significantly more Axis I disorders and Axis II disorders, as well as alcohol and drug abuse. Of note, the number of suicide attempts was not significantly different between groups and age periods.

Even though the results were not significant, both groups were exposed to antecedents of suicide death: 26% of individuals in the suicide group and 17% of the control group had histories of suicide among first-degree family members. However, when we cumulate number of first-degree family members, extended family members, and friends, 56% of the suicide group were exposed to one to three suicide deaths, and 8.6% were exposed to between four and nine suicides. As for the control group, 65% were exposed to one to three suicide deaths, and 5.5% were exposed to between four and nine suicides in their lifetime (Table 5).

## 4. Discussion

Results from this study indicate significant differences between living-control and suicides-cases, resulting in a lower burden of adversity among the control-group throughout the life course. Though the control-group was exposed to adversities, they were not exposed to the severity of adversity compared to the suicide-group. In a recent study from our research group with the same participants [32], findings demonstrated that people with no suicide attempt had more protective variables throughout their lifespan than people who died by suicide. More specifically, the protective factors offered by the social environment show the greatest difference, indicating that universal strategies that could have an impact on increasing community connectedness and stability may have a positive impact on individual suicide vulnerability.

As for people included in the suicide group, they were significantly more exposed to early adversity and were exposed to a higher level of adversity in different spheres of life (e.g., early adversity, family environment, personal and mental health sphere, social sphere, etc.). Early adversity, such as psychological and physical violence and household dysfunction during early childhood, may impact on the future course of development and promote the emergence of additional problems that can contribute to vulnerability to suicide, including aggressiveness, impulsivity, behavior dysregulation, inefficient coping styles [42,43], poor attachment skills [44], and lack of personal control or mastery over their lives [45]. This observation has been abundantly confirmed in many fields and in research conducted with many different populations in the last few decades [30,46,47,48,49,50].

Results from this study report that 86% of individuals in the suicide group were exposed to at least one adverse household and family experience, compared to 67% of individuals of the control group. The cumulative effect of adverse events may have an impact on the overall burden of adversity and lead to suicide vulnerability over time. The epidemiological ACE (Adverse Childhood Experience) study in the US population [51] showed that the likelihood of suicide attempt was found to be more than 12 times greater for those who had four ACEs than for those who reported no adverse childhood experiences. This developmental pattern suggests that early adversity (e.g., being witness to family tension and violence, parental mental health problems, neglect, intimidation, etc.) coupled with socioeconomic disadvantage (e.g., overcrowding and food insecurity in the household), represents an important risk factor for a cascading effect leading to the presence of early mental health difficulties, such as alcohol and substance abuse in young adolescents, and eventually social and relational problems contributing to suicide vulnerability. Our findings affirm the importance of selective interventions targeting children, youth, and young adults who are exposed to violence and/or psychologically dysfunctional households.

### Limitations

One important limitation is the possible lack of cultural perspective. There is a long and painful history of research focusing upon the individual and social deficits in Canadian Inuit culture and society. Along with other aspects of the colonial enterprise, this research may contribute to inaccurate beliefs about Inuit culture in the dominant society and undermine the pride that Inuit have in their culture and heritage [52]. This limitation can also deflect attention from the many positive individual, environmental, and social forces within Inuit culture and society, which have been found to help protect against suicide [32]. When considering the current study, readers should keep in mind that the strength, resilience, and adaptability of Inuit far outweigh the vulnerabilities discussed in this article, as witnessed by the tremendous advancements that have been made in the face of great historical and contemporary disadvantage.

Other limitations relate to the use of the retrospective method. First, the reliability of recall of events is uncertain. Although every effort was made to maximize the accuracy of the retrospective reports and construct an accurate timeline, there may have been important events that close relatives or friends were not privy to. A large number of variables were tested as potential predictors, but robust statistical methods were used to ensure statistical validity. Worth noting is the effort made throughout this methodology to maximize the accuracy of retrospective reports and effectively record the timeline of events and the onset of mental health problems. Although the narrative methodology has limitations, it is consistent with the way in which personal memory is organized and, hence, more likely to elicit potential causal factors than methods that elicit facts in isolation. 

Lastly, with the exception of substance abuse disorder, mental health problems were not described in detail, except to identify the age period in which the participant acknowledged the difficulties started. The small number of participants made it difficult to find statistical significance between age period and type of disorders. However, the analysis did show the burden of mental health problems and alcohol and drug abuse on suicide vulnerability. Axis I and Axis II disorders could be a factor in suicide vulnerability and should be considered in greater depth in future research.

## 5. Conclusions

This is the first case-control study to trace the life trajectories of Inuit in Nunavut who died by suicide. Our results clearly indicate a heavier burden of adversity among individuals who died by suicide, starting early in life and creating a cascade of risk. While results indicate that the types of risk factors are similar to those found in literature on suicide, the overall burden of adversity for Inuit is more important and may result from different sources, such as the many historical and contemporary traumas that resulted from colonization. It is important to note that the load of the risk factors stemmed from determinants of health factors, such as overcrowded housing and food insecurity. Such factors can create stress, tensions, and difficulties in parental relations and parental roles, resulting in children being exposed to higher levels of adversity within the household. Taking action to improve the conditions of daily life during early childhood and school age would provide upstream opportunities, both to improve the population’s mental health and to reduce the risk of suicide.

## Figures and Tables

**Table 1:** Trajectory parameters.

	Living	Suicide
Percentage of sample	50.0%	50.0%
Parameters, means (SE)		
Intercept	2.857 (0.122) ***	4.077 (0.118) ***
Linear change	0.261 (0.078) ***	0.324 (0.110) **
Quadratic change	0.046 (0.016) **	0.022 (0.024) NS

** *p* < 0.01, *** *p* < 0.001.

**Table 2:** Early maltreatment: types and number of abuse among groups in the five-to-nine age period.

	Type of Abuse				Number of Abuse			
	Physical	Sexual	Psychological	Incest	0	1	2	3
Suicide group	16 (17.4%)	2 (2.2%)	19 (20.7%) *	6 (6.5%)	61 (66.3%)	21 (22.8%)	8 (8.7%)	2 (2.2%)
Living	12 (13.0%)	3 (3.3%)	7 (7.6%) *	0 (0.0%)	76 (82.6%)	10 (10.9%)	6 (6.5%)	0 (0.0%)

* *p* < 0.05. ϕ (phi) value for the psychological abuse difference = 0.19.

**Table 3:** Number of variables present for each sphere: comparison between groups.

Sphere	Themes	Nb Items	Suicide		Living		*p* (ϕ)
			Maximum	% Zero	Maximum	% Zero	
			Count		Count		
Early adversity of maltreatment	Subjected to sexual, physical or psychological violence *	4	3	66.3	2	82.6	<0.05 (0.19)
Household and family Dysfunction	Conflictual or dysfunctional family environment **	17	8	14.1	7	32.6	<0.01 (0.22)
Personal/mental health difficulties	Mental health problems ***	9	3	53.3	3	82.6	<0.01 (0.31)
Social difficulties	Conflicts with peers, isolation, bad influence ****	5	5	75.0	5	89.1	<0.05 (0.18)

* Early adversity of maltreatment included variables such as incest, sexual violence, physical violence, and psychological violence. ** Household and family dysfunction included variables such as incoherent rules, tension, lack of discipline, negligence, role reversal, affective distance, family secrets, parental separation, parental divorce, parental mental health difficulties, witnessing parental violence, living with someone who misuse alcohol, tension with sibling, overcrowding, lack of supplies, difficult housing conditions, food shortage, etc. *** Personal/mental health difficulties included variables such as Axis I diagnosis, personality disorder, alcohol and drug abuse, number of suicide attempts, other problems of mental health, and psychiatric hospitalization. **** Social difficulties included variables such as conflict with peers, social isolation, bad influence from peers, resulting in legal problems, separation or loss of a friend, and difficulty in relationship commitment. Φ is the phi value.

**Table 4:** Diagnoses and suicide attempts by age and groups.

	Total		Living		Suicide		χ2 (ϕ)	OR	CI95%	*p*
	(*n* Initial = 184)		(*n* = 92)		(*n* Initial = 92)					
	*n*/*n* Alive	%	*n*	%	*n*/*n* Alive	%				
Axis I (5–9 years)	8/184	4.34	2	2.17	6/92	6.52	-	-	-	NS
Axis I (10–14 years)	25/181	13.8	3	3.26	22/89	24.7	17.5 (0.31)	9.7	3.2–38	<0.001
Axis I (15–19 years)	77/149	50.3	20	21.7	55/57	96.5	78.7 (0.73)	99	23–448	<0.001
Axis II (5–9 years)	0/184	0	0	0	0/92	0	-	-	-	NS
Axis II (10–14 years)	3/181	1.65	0	0	3/89	3.37	-	-	-	NS
Axis II (15–19 years)	19/149	12.8	4	4.34	15/57	26.3	15.3 (0.32)	7.8	12–28	<0.001
Alcohol/Drug abuse	6/184	3.26	2	2.17	4/92	4.35	-	-	-	NS
(5–9 years)										
Alcohol/Drug abuse	35/181	18.8	9	9.78	25/89	28.1	9.9 (0.23)	3.6	1.6–8.1	<0.005
(10–14 years)										
Alcohol/Drug abuse	82/149	51.7	34	37.1	43/57	75.4	20.9 (0.37)	5.2	2.4–10	<0.001
(15–19 years)										
Number of suicide attempts										
0	111	60.3%	58	63.0%	53	57.6%	-	-	-	NS
1	35	19.0%	17	18.5%	18	19.6%	-	-	-	NS
2	14	7.6%	5	5.4%	9	9.8%	-	-	-	NS
3+	24	13.0%	12	13.0%	12	13.0%	-	-	-	NS

(ϕ) Corresponds to the phi value.

**Table 5:** History of suicide completion among first-degree family members and friends.

	First-Degree Family Member(s)		*n*	Cumulative Numbers of First-Degree, Extended Family Member(s) and Friend(s)		
**History of Suicide Completion**	**0**	**1 to 3**		**0**	**1 to 3**	**4 to 9**
Suicide group	68 (73.9%)	24 (26.1%)	92	32 (34.8%)	52 (56.6%)	8 (8.6%)
Living	76 (82.6%)	16 (17.4%)	92	27 (29.3%)	60 (65.2%)	5 (5.5%)

First-degree family member: mother, father, sister(s), or brother(s).
